# Shikonin inhibits proliferation of melanoma cells by MAPK pathway-mediated induction of apoptosis

**DOI:** 10.1042/BSR20203834

**Published:** 2021-01-22

**Authors:** Jae Han Lee, So Hee Han, You Min Kim, Sung Hyun Kim, Eun Seon Yoo, Joong Seok Woo, Gi Hwan Jung, Soo Hyun Jung, Bum Seok Kim, Ji Youn Jung

**Affiliations:** 1 Department of Companion and Laboratory Animal Science, Kongju National University, Yesan, Republic of Korea; 2 College of Veterinary Medicine and Bio-safety Research Institute, Jeonbuk National University, Iksan, Republic of Korea

**Keywords:** apoptosis, Melanoma cells, mitogen-activated protein kinases, Shikonin

## Abstract

Shikonin, a natural product isolated from the roots of *Lithospermum erythrorhizon*, exhibits pharmacological effects against inflammation, ulcers, infections, and tumors. In the present study, we aimed to investigate the antitumor effects of shikonin on the human melanoma cell line, A375SM, and in an *in vivo* mouse xenograft model. We examined the anticancer effects of shikonin by *in vitro* experiments (MTT (3-(4,5-dimethylthiazol-2-yl)-2,5-diphenyltetrazolium bromide) assay, 4′,6-diamidino-2-phenylindole (DAPI) stain, annexin V/ propidium iodide (PI) stain, and protein analysis of apoptosis and mitogen-activated protein kinase (MAPK) pathways). Further, the anticancer effect *in vivo* was confirmed through a xenograft model. Our results showed that shikonin inhibited the proliferation of melanoma cells in a dose-dependent manner. In addition, shikonin significantly increased nucleus and chromatin condensation and early/late apoptosis. Shikonin also increased the pro-apoptotic proteins and decreased the anti-apoptotic proteins. Additionally, shikonin was overexpressed in MAPK pathways. Investigation of the effects of shikonin in a mouse xenograft model not only showed decreased A375SM tumor volume but also increased apoptosis as determined by terminal deoxynucleotidyl transferase dUTP nick-end labeling (TUNEL) assay. Furthermore, pathologic changes were not observed in the liver and kidney of mice. Collectively, the study indicated that shikonin inhibited the proliferation of the human melanoma cells by inducing apoptosis, mediated by MAPK pathway and that it is a potential candidate for an anticancer drug against melanoma cancer.

## Introduction

Melanoma is a type of skin cancer that begins in melanocytes and accounts for 10% of all skin cancer cases worldwide. Although it is less common than other types of skin cancer, melanoma is associated with high fatality as it accounts for more than 75% of deaths from skin cancer [1]. Risk factors for melanoma include excessive exposure to ultraviolet (UV) rays of the sun, which results in DNA damage in melanocytes located in the basal layer of the epidermis, weakened immune system, and dysplastic nevi [2,3]. Currently, treatment options for melanoma include surgery, chemotherapy, radiation therapy, and immunotherapy, and early-stage melanomas can be successfully treated in most cases with surgical therapy. However, these therapies are not very effective in the treatment of unresectable melanomas, or metastatic melanomas, owing to their high resistance to the currently available chemotherapy and radiation therapy [4,5]. Moreover, both therapies are associated with a number of side effects, as normal cells are also affected during the process [6,7]. Therefore, development of novel therapeutics with fewer side effects is warranted. Studies are currently underway to develop anticancer agents from natural products to reduce undesirable side effects [8–10].

**Figure 1:**
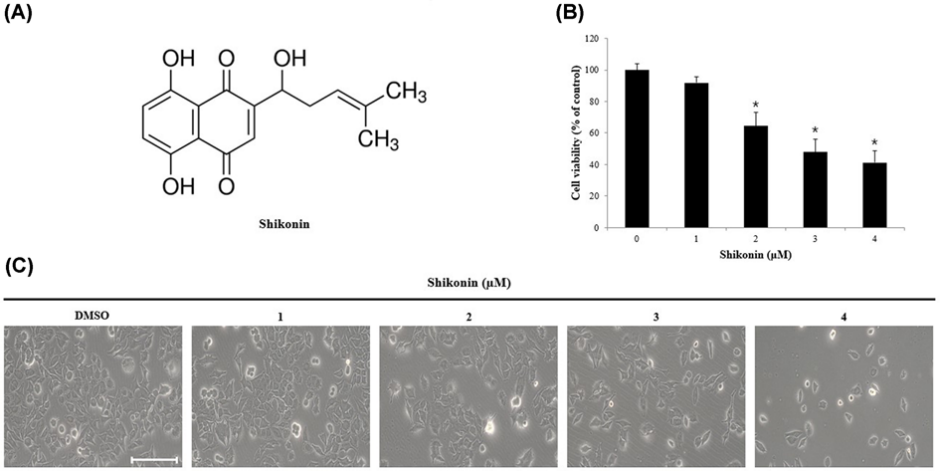
Effect of shikonin in human melanoma cell (**A**) Chemical structure of shikonin. **(B**) A375SM cells were seeded in 96-well plates, incubated for 24 h and then treated with various concentrations of shikonin for 24 h. Cell viability was measured by MTT assay. (**C**) Morphologic changes in A375SM cells treated with various concentrations of shikonin for 24 h were analyzed under a light microscope (scale bar, 10 μm) Control cells were treated with DMSO. Data are presented as mean and standard deviation (SD) for three samples. Significance was determined by Student’s *t* test, *P<0.05, compared with the control. Abbreviations: DMSO, dimethylsulfoxide; MTT, 3-(4,5-dimethylthiazol-2-yl)-2,5-diphenyltetrazolium bromide.

Shikonin (Figure 1A) is a natural product isolated from *Lithospermum erythrorhizon*, which belongs to the family Boraginaceae, and it has long been used in the treatment of inflammation, burns, ulcers, infections, and cancer in China [11,12]. Previous studies have reported the anticancer effects of shikonin, including its involvement in p27 and p53 regulation and cytochrome *c* release in colon cancer [13], in addition to the induction of apoptosis via extracellular signal-regulated kinase (ERK)1/2 and c-Jun N-terminal kinase (JNK) in leukemia [14,15].

Apoptosis is a form of programmed cell death, protecting an organism by removing damaged, virus-infected, or cancerous cells, and includes processes such as cytoplasmic shrinking, extensive plasma membrane blebbing, and nuclear condensation [16,17]. Apoptotic pathways may be classified into the mitochondrial-mediated intrinsic and cell death receptor-mediated extrinsic pathways; caspases generated by the combined action of these two pathways actively lead to apoptosis. Key apoptosis inhibitors include Bcl-2 and Bcl-xL, whereas key inducers include Bcl2 (B-cell lymphoma-2)-associated X protein (Bax), Bid, and Bim [18,19]. The activation and function of caspases, involved in the caspase-cascade system, are regulated by various molecules, such as the apoptotic inhibitor proteins, Bcl-2 family proteins, calpain, and Ca^2+^ [20]. In addition, caspase-3 mediates the cleavage of poly (ADP-ribose) polymerase (PARP) during cell death [21]. Loss of apoptotic control in cells allows them to proliferate and become cancerous; conversely, unregulated apoptosis leads to degenerative diseases [22]. Therefore, investigations are underway to identify anticancer compounds with apoptosis inducing and regulating effects, so that they may be applicable for cancer treatment [23–25]. Additionally, mitogen-activated protein kinases (MAPKs) are serine/threonine kinases that are involved in cellular response to stress, differentiation, proliferation, and apoptosis [26]. The major MAPK pathways include ERK1/2, JNK, and p-38 MAPK pathways, and are responsive to various cell stimulations. ERK1/2 pathways are involved in cell proliferation and survival, whereas JNK and p-38 pathways are responsive to stress such as DNA damage, osmolarity changes, and chemotherapeutic drugs [27–29]. One of the major consequences of the exposure of p-38 MAPK signaling to stress is apoptosis. p-38 MAPK induces apoptosis through two different mechanisms, promoting the transcription of pro-apoptotic genes and direct activity of Bcl-2 family proteins [30].

Although the anticancer effect of apoptosis induced by p53 regulation in A375-S2 melanoma cells has been reported previously [31], few studies have investigated the induction of apoptosis via MAPK pathway and its anticancer effect in A375SM melanoma cells. Therefore, in the present study, *in vitro* experiments were conducted to examine the inhibitory effect of shikonin on the proliferation of A375SM melanoma cells and to determine whether the inhibition of proliferation was mediated by the MAPK apoptotic pathway. Furthermore, *in vivo* experiments were performed to study the effects of shikonin in physiological systems.

## Methods

### Reagents and cell lines

Dulbecco’s modified Eagle’s medium (DMEM), purchased from Welgene (Gyeongsan, Korea), was used for cell culture, and fetal bovine serum (FBS) and penicillin were purchased from Gibco BRL (Grand Island, NY, U.S.A.). A375SM melanoma cells (catalog no. 80004) were purchased from Korea Cell Line Bank (KCLB). Cancer cells were cultured in DMEM containing FBS (5%) and penicillin/streptomycin (1%), at 37°C under 5% CO_2_. Other general reagents, such as 3-(4,5-dimethylthiazol-2-yl)-2,5-diphenyltetrazolium bromide (MTT) and 4′,6-diamidino-2-phenylindole (DAPI), were purchased from Sigma–Aldrich Co. (St. Louis, MO, U.S.A.). Fluorescein Isothiocyanate (FITC)-Annexin-V detection kit (catalog no. 556420) was purchased from BD Pharmingen™ (San Diego, CA, U.S.A.). Primary antibodies for Bax (rabbit, 1:1000, #2772), Bcl-2 (rabbit, 1:1000, #4223), PARP (rabbit, 1:1000, #9542), ERK1/2 (rabbit, 1:1000, #9102), p-ERK1/2 (rabbit, 1:1000, #9101), JNK (rabbit, 1:1000, #9252), p-JNK (rabbit, 1:1000, #4668), p38 (rabbit, 1:1000, #9212) and phosphorylated p38 (p-p38; rabbit, 1:1000, #9211), and secondary antibodies for rabbit IgG (rabbit, 1:1000, #7074) were purchased from Cell Signaling Technology (Beverly, MA, U.S.A.), and β-actin (mouse, 1:1000, sc-47778) and mouse IgG (mouse, 1:1000, sc-516102) were purchased from Santa Cruz Biotechnology Inc. (Dallas, TX, U.S.A.). Shikonin was purchased from Sigma–Aldrich Co. (St. Louis, MO, U.S.A., purity > 98% as determined by HPLC) and dissolved in dimethylsulfoxide (DMSO), and stored at −20°C.

### MTT assay

MTT assay was performed to examine the inhibitory effect of shikonin on melanoma cells viability. A375SM melanoma cells were plated on 96-well plates at 2 × 10^4^ cells/ml and incubated for 24 h. The cells were then treated with different concentrations of shikonin (0, 1, 2, 3, and 4 μM) for 24 h. Next, 40 μl of MTT solution (5 mg/ml) was added into the wells of 96-well plates containing cells treated with shikonin for 24 h, and the plates were incubated for 2 h at 37°C under 5% CO_2_. After incubation, the MTT reagent was removed, and the cells were treated with DMSO (100 μl/well) to completely dissolve the formazan product formed in the wells. Absorbance was measured at 595 nm using an ELISA reader (Bio-Rad Laboratories Inc., Hercules, CA, U.S.A.).

### DAPI staining

DAPI was performed to examine the morphological changes in the nucleus that specifically occur upon the induction of apoptosis. A375SM cells were plated on 60-mm dishes at 1 × 10^5^ cells/ml and kept undisturbed for 24 h to ensure proper adhesion. The cells were then treated with different concentrations of shikonin (0, 2, and 4 μM) and incubated for another 24 h. Thereafter, the cells were washed with PBS and fixed with 4% formaldehyde solution for 15 min. The cells were washed again with PBS, and 2 ml of DAPI reagent was added. The cells were examined under a fluorescence microscope (Zeiss Fluorescence Microscope, Thornwood, NY, U.S.A.) at 200× magnification. DAPI-positive cells were counted in one microscopic field and expressed as a percentage of the total number of cells.

### Flow cytometry analysis

Annexin V/propidium iodide (PI) staining was performed to quantitatively analyze the level of apoptosis induced by shikonin in melanoma cells. A375SM cells were treated with different concentrations of shikonin (0, 2, and 4 μM) and incubated for 24 h. The cells were detached using trypsin-EDTA and were centrifuged (290×***g***) to obtain cell pellets. The pellets were washed with PBS and centrifuged once again; the resulting cell pellets were suspended in 1× binding buffer up to a concentration of 2 × 10^5^ cells/ml, and were incubated with annexin-V and PI for 15 min. The cells were analyzed using the FACSCalibur™ flow cytometer (BD Biosciences, NJ, U.S.A.).

### Western blotting

Western blotting was performed to determine the expression of apoptosis-related proteins. A375SM cells were incubated in 75T flasks for 24 h at 37°C under 5% CO_2_, and then treated with different concentrations of shikonin (0, 2, and 4 μM). The cells were detached with trypsin-EDTA and centrifuged. After washing with PBS, the cells were centrifuged again, followed by the addition of cell lysis buffer (Invitrogen, CA, U.S.A.) to the pellets and incubation of the same for 20 min at 4°C. The lysates were centrifuged and supernatants were collected for use in the experiments. The concentration of extracted protein was measured using the Bradford protein assay. Extracted proteins were separated by electrophoresis in 12% sodium dodecyl sulfate/polyacrylamide gel and then transferred to nitrocellulose membranes. After protein transfer, the membranes were blocked in 5% skim milk for 2 h and incubated overnight at 4°C with primary antibodies (diluted 1:1000). The membranes were subsequently incubated with secondary antibodies (diluted 1:1000), including rabbit IgG or mouse IgG antibodies, for 2 h. Each protein band was examined using enhanced chemiluminescence (ECL)-detection reagents (Pierce, Rockford, IL, U.S.A.), and the density was measured using ImageJ Launcher (provided by NCBI).

### Xenograft establishment

BALB/c nude male mice (18–22 g, 4 weeks old) were purchased from Nara Biotech (Seoul, Korea). The use of animals in the present study was approved by the Institutional Animal Care and Use Committee of Kongju National University (KNI_2020-2, Chungcheongnam-do, Korea). All *in vivo* experiments were performed in Kongju National University (Chungcheongnam-do, Korea) in accordance with relevant guidelines and regulations. The mice were housed under a 12-h day/night cycle at 23 ± 3°C and 50 ± 10% humidity. For xenograft, A375SM cells were injected into each shoulder of the mice at the concentration of 5 × 10^6^ cells/ml in PBS. After tumor formation, the mice were divided into two groups (*n*=5), and injected with shikonin (0 and 4 mg/kg) intraperitoneal every 3 days. Tumor volume was measured every 3 days using a Vernier caliper (Mitutoyo Corporation), and tumor volume was calculated using the formula: volume = {(width + length) × 0.5}³. All mice were killed using CO_2_ gas (30% per min, 3 min) at the end of 30 days, and tumors were excised and weighed.

### Hematoxylin and Eosin staining

After fixing the liver and kidney in 10% formaldehyde, paraffin blocks were prepared and 5-μm sections were prepared. Tissue sections were stained with Hematoxylin and Eosin (H&E) and examined at 200× magnification under a light microscope.

### TUNEL assay

Terminal deoxynucleotidyl transferase dUTP nick-end labeling (TUNEL) assay was performed to determine whether shikonin administration induced apoptosis in tumor cells. First, 5-μm tissue sections were deparaffinized with xylene and hydrated with ethanol. Each slide was then washed with PBS and incubated with 100 μl of proteinase K (20 μg/ml) for 15 min at room temperature. Thereafter, equilibration buffer and biotinylated nucleotide mix was mixed with rTdT, and 100 μl of the mixture was added to each slide, followed by incubation for 1 h at 37°C. Next, endogenous peroxidase was blocked by incubating the slides with a mixture of 0.3% H_2_O_2_ and PBS for 5 min, followed by incubation with HRP–conjugated streptavidin for 30 min at room temperature. After washing with PBS, the slides were incubated with 3,3′-diaminobenzidine tetrahydrochloride (DAB) solution for 10 min, mounted, and examined through light microscopy (200×).

### Immunohistochemistry

Immunohistochemistry was performed to determine the expression of apoptosis-related proteins in tumor cells. First, 5-μm tissue sections were deparaffinized with xylene and hydrated with ethanol. Then, the sections were washed with PBS, and endogenous peroxidase was blocked with 0.3% H_2_O_2_. After washing with PBS, blocking was performed with skimmed milk, and the sections were incubated overnight with primary antibodies (anti-p-p38, diluted 1:100) at 4°C. After a washing step, the sections were incubated with secondary antibodies (rabbit-IgG, 1:100) at room temperature for 2 h, followed by incubation with DAB and H_2_O_2_. Subsequently, the sections were stained with Hematoxylin, mounted, and examined by light microscopy (200×).

### Statistical analysis

All experimental results are expressed as mean ± standard deviation. Comparisons between groups were done using ANOVA, followed by *t* test analysis. Results that differed from the control group with *P*<0.05 were considered statistically significant.

## Results

### Effects of shikonin on viability of A375SM melanoma cells

MTT assay was performed to determine the effects of shikonin on melanoma cells viability. After treatment with 1, 2, 3, and 4 μM shikonin for 24 h, the viability of A375SM cells was 91, 64, 47, and 41%, respectively, compared with that of the DMSO-treated control, showing a decrease with increasing concentration of shikonin. Treatment with concentrations exceeding 2 μM shikonin resulted in a significant reduction in cell viability (Figure 1B). After treatment with shikonin, morphologic changes were also observed, including cytoplasmic shrinkage and membrane blebbing (Figure 1C).

### Examination of morphological changes in A375SM melanoma cells upon shikonin treatment

**Figure 2:**
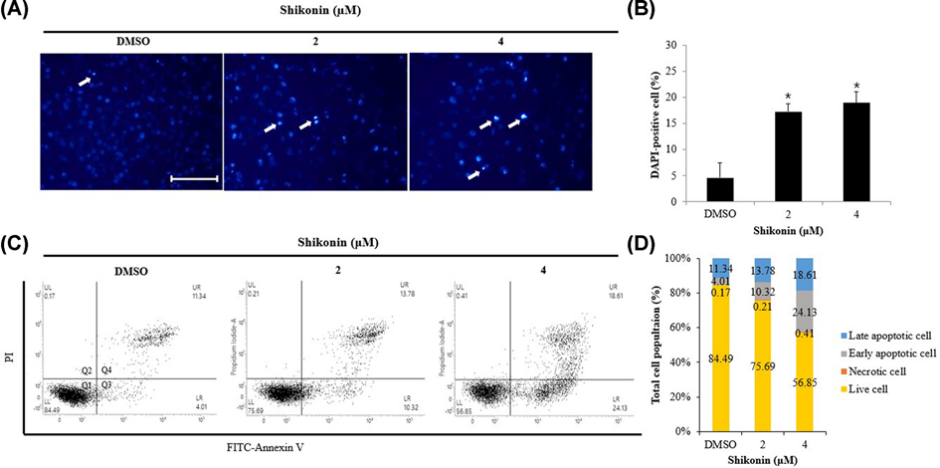
Induction of apoptosis in melanoma cells upon treatment with shikonin (**A**) A375SM cells were seeded for 24 h and then incubated with indicated concentrations of shikonin for 24 h. Cells were fixed and stained with 1× DAPI solution. Cell morphological characteristics were analyzed using a fluorescence microscope (scale bar, 10 μm). (**B**) The bar graph represents the average of four fields under a fluorescence microscope, and the percentage of DAPI-positive cell among all cells. (**C**) A375SM cells were seeded for 24 h. After treating with the indicated concentration of shikonin for 24 h, the cells were collected and stained with Annexin-V/PI, and then analyzed by flow cytometry. (**D**) Bar graph depicting the percentage of live, necrotic, early, and late apoptotic cells. (Q1: live, Q2: necrotic, Q3: early apoptotic, Q4: late apoptotic). Data are presented as mean and standard deviation (SD) for three samples. Significance was determined by Student’s *t* test, *P<0.05, compared with the DMSO-treated control.

To determine whether the decrease in cell viability was due to apoptosis, A375SM cells were treated with 0, 2, and 4 μM shikonin for 24 h, and nuclei were stained with DAPI. Fluorescence microscopy showed no remarkable change in the control cells, although many DAPI-positive cells were observed in the shikonin-treated cells (Figure 2A). Quantification of DAPI-positive cells revealed 18% in the 4 μM shikonin-treated cells, which was significantly higher compared with 4% DAPI-positive cells of the control group (Figure 2B).

### Induction of apoptosis in A375SM melanoma cells upon shikonin treatment

DAPI staining suggested that the decreased viability of cancer cells after shikonin treatment may be attributed to apoptosis. Hence, early apoptosis and late apoptosis rates were measured by flow cytometry following annexin-V/PI staining (Figure 2C). Early apoptosis rate increased from 4.01 to 10.32%, and then to 24.13%, with increasing shikonin concentration. Late apoptosis rate was 11.34, 13.78, and 18.61% for the 0, 2, and 4 μM-treated cells, respectively, thereby demonstrating that shikonin treatment increases both early and late apoptosis rates (Figure 2D).

### Examination of apoptosis-related protein expression in A375SM melanoma cells upon shikonin treatment

**Figure 3:**
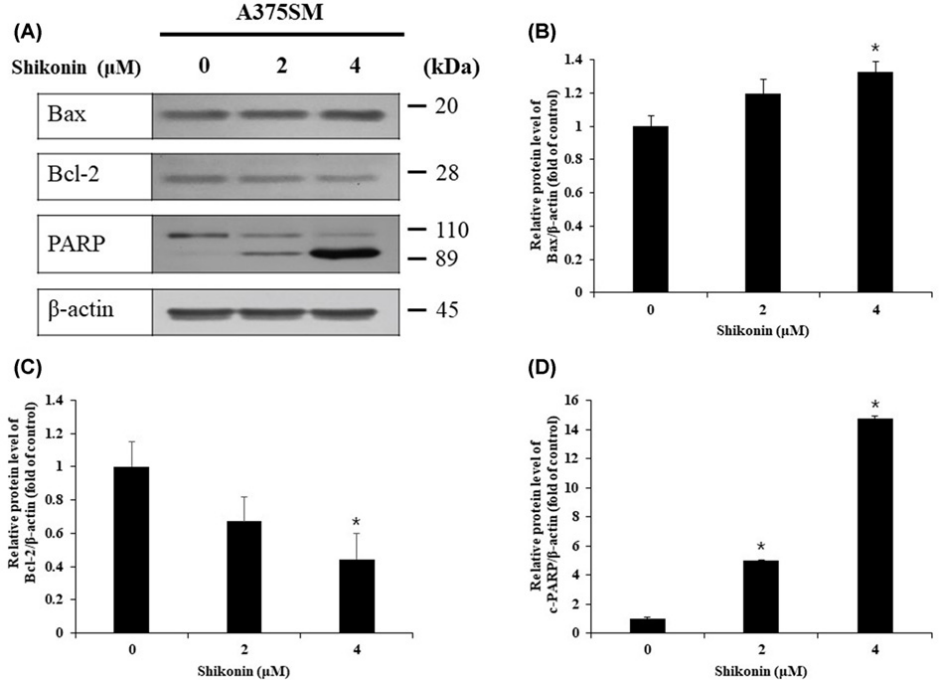
Western blot analysis of apoptosis-related proteins in melanoma cells (**A**) A375SM cells were treated with indicated concentrations of shikonin for 24 h and harvested to measure expression levels of Bcl-2, and activation of Bax and cleaved-PARP were detected by Western blot analysis. (**B–D**) Bar graph was generated by quantifying blots from three independent experiments using ImageJ and normalizing the intensity of the bands to the DMSO-treated control. Significance was determined by Student’s *t* test, *P<0.05, compared with the DMSO-treated control.

Western blotting was performed to determine the expression level of apoptosis-related proteins. The expression of Bax, a key inducer of apoptosis, was higher in the shikonin-treated cells compared with the control cells. The expression of Bcl-2, an inhibitor of apoptosis, tended to decrease in the shikonin-treated cells compared with that in the control cells. Fragmentation was observed through increased expression of the cleaved fragment of PARP, which is known to be involved in DNA repair and is a caspase substrate (Figure 3).

### Induction of apoptosis via MAPK pathway in A375SM melanoma cells upon shikonin treatment

**Figure 4:**
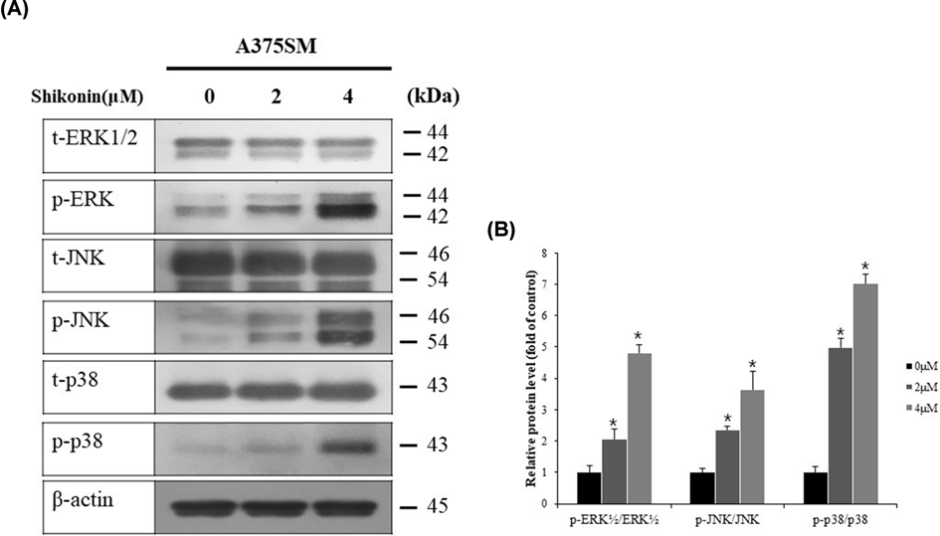
Western blot analysis of MAPK pathway in melanoma cells (**A**) A375SM cells were treated with indicated concentrations of shikonin for 24 h and harvested to measure expression levels of MAPK pathways; ERK, JNK, and p38 proteins were detected by Western blot analysis. (**B**) Bar graph was generated by quantifying blots from three independent experiments using ImageJ and normalizing the intensity of the bands to the DMSO-treated control. Significance was determined by Student’s *t* test, *P<0.05, compared with the DMSO-treated control.

Western blotting was performed for ERK, JNK, and p38 proteins, which are major participants in the MAPK pathway, to determine the underlying mechanism of apoptosis induction. An increased expression of phosphor-ERK1/2, phosphor-JNK, and phosphor-p38 was observed in the shikonin-treated cells compared with that in the control group. Total-ERK1/2, total-JNK, and total-p38 tended to decrease in the shikonin-treated cells (Figure 4A). Quantification of these proteins showed that the level of phosphorylation of these proteins increased with increasing concentrations of shikonin (Figure 4B).

### Effects of shikonin on xenograft tumors

**Figure 5:**
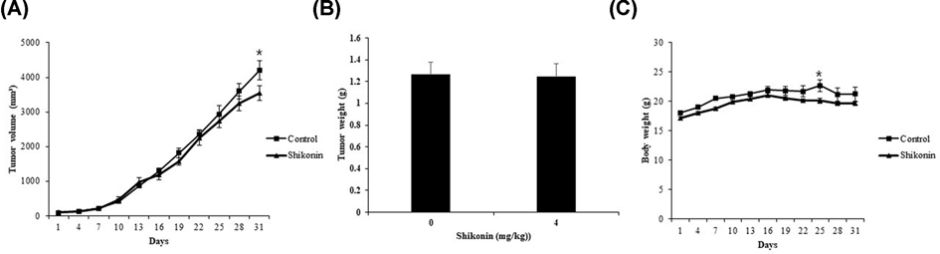
Effects of shikonin on melanoma tumor growth Nude mice bearing A375SM cells as a xenograft model were treated with shikonin (4 mg/kg) for 30 days, and (**A**) tumor volume, (**B**) weight, and (**C**) body weight were determined. Each value represents the mean ± SE. Student’s *t* test (*P<0.05, control animals were treated with PBS consisting of 0.1% DMSO).

To determine the *in vivo* effect of shikonin on tumors, we utilized a mouse xenograft model. A375SM cells were cultured and subcutaneously injected into both shoulders of mice. After confirming tumor formation, the mice were divided into two groups (0 and 4 mg/kg shikonin) [32]. Shikonin administration was initiated once the tumor attained a volume of approximately 90 mm³. Intraperitoneal administration of shikonin, once every 3 days, was performed for a total of 30 days. A decreased tumor volume was observed (Figure 5A), although there was no significant reduction in tumor weight, as per measurements following autopsy (Figure 5B). No significant reduction in weight was observed in the shikonin-treated mice compared with that of the control group (Figure 5C).

### Induction of apoptosis in melanoma tumors by shikonin administration

**Figure 6:**
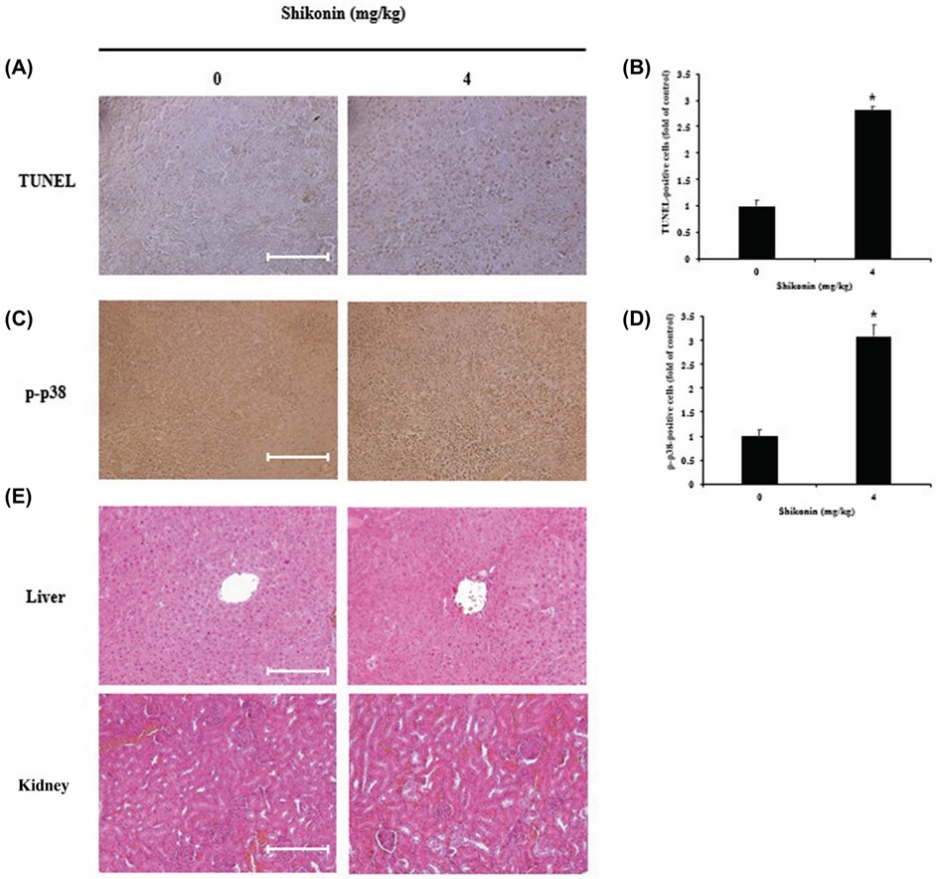
Shikonin induced apoptosis in melanoma tumor tissues (**A**) Apoptosis was measured in tumor tissues using the TUNEL assay and (**C**) phospho-p38 expression was measured in tumor tissues by immunohistochemistry. (**B**) TUNEL-positive and (**D**) p-p38-positive cells were observed under a light microscope and are shown as the average of four fields. Significance was determined by Student’s *t* test, *P<0.05, compared with the untreated control. (**E**) Histological toxicity analysis of the liver and kidney in nude mice by H&E staining. Slides were observed under a light microscope (scale bar, 10 μm).

TUNEL assay was performed to determine whether shikonin induced apoptosis in melanoma tumors in mice. Results showed no significant change in the control mice, although TUNEL-positive cells were observed in the shikonin-treated mice (Figure 6A). Quantification of these TUNEL-positive cells indicated that their abundance was approximately 2.8-times more in the shikonin-treated mice compared with that in the control group (Figure 6B).

### Effects of shikonin administration on apoptosis-related proteins in melanoma

Immunohistochemical staining was conducted to determine the effects of shikonin on apoptosis-related proteins in melanoma. Examination of p-p38, a key protein in the MAPK pathway, showed that its expression was clearly increased in the shikonin-treated mice, compared with that in the control group (Figure 6C). Quantification of p-p38-positive cells revealed that the cells were approximately 3.0-times more abundant in the shikonin-treated mice compared with that in the control mice (Figure 6D).

### Examination of histopathological changes in the liver and kidney after shikonin administration

H&E staining was performed to determine whether shikonin administration induced toxicity in the liver and kidney of mice. No significant difference in the liver and kidney (Figure 6E) was observed in mice from the shikonin-treated group and control group.

## Discussion

Shikonin is a flavonoid found in *L. erythrorhizon*, a perennial herbaceous plant belonging to the family Boraginaceae, and is known to have physiological effects, particularly therapeutic effects on inflammation, burns, and infections [11,12]. It has also been reported to exhibit anticancer effects on several cancers [13–15]. However, few studies have investigated its anticancer effect on melanoma. Therefore, the present study investigated the inhibitory effect of shikonin on the proliferation of A375SM melanoma cells and determined whether the anticancer effect was mediated by apoptosis.

The effect of shikonin on melanoma cells viability was determined by MTT assay of A375SM cells treated with 1, 2, 3, and 4 μM shikonin, which does not affect normal cells [33], for 24 h. Results showed a significant reduction in viability upon treatment with more than 2 μM shikonin (Figure 1B). Induction of apoptosis is characterized by morphological changes like apoptotic bodies, including chromatin and nuclear condensation resulting from DNA fragmentation and cell membrane bubbling [34]. Therefore, to determine whether the inhibitory effect of shikonin on the proliferation of melanoma cells was due to apoptosis, DAPI staining was performed. A375SM cells, treated with 2 and 4 μM shikonin, showed an increased number of apoptotic bodies, a characteristic feature of apoptosis (Figure 2A). Quantification of the DAPI-positive cells revealed that the percentage of apoptotic bodies in the cells of the treatment groups was higher (4.50, 17.28, and 18.95%) than that in the control group, and increased in a shikonin concentration-dependent manner (Figure 2B).

During the early stages of apoptosis, nuclear condensation and cell membrane bubbling occur simultaneously, resulting in the translocation of phosphatidylserine (PS) from the inner leaflet to the outer leaflet of the cell membrane. Annexin-V binds to PS and allows the staining of early and late apoptotic cells [35]. In contrast, PI primarily attaches to the nucleus, selectively staining late apoptotic and necrotic cells, rather than live cells or early apoptotic cells [36]. To measure the rate of apoptosis, A375SM cells were treated with 0, 2, and 4 μM shikonin and stained with annexin-V and PI (Figure 2C). The total amount of apoptotic cells (the sum of early and late apoptotic cells) increased with increasing shikonin concentration (15.35, 24.10, and 42.74%), and the differences were significant (Figure 2D). Therefore, shikonin inhibited the proliferation of A375SM melanoma cells, possibly due to induction of apoptosis.

Bcl-2 family proteins are important for apoptosis regulation, and comprise pro-apoptotic proteins, such as Bax, Bad, and Bid, and anti-apoptotic proteins, such as Bcl-2 and Bcl-XL. Apoptosis regulation is dependent on the ratio of pro-apoptotic to anti-apoptotic proteins [37]. Furthermore, PARP is a nuclear protein involved in DNA repair, and is activated by DNA damage. Caspase activation induces apoptosis and leads to fragmentation of PARP, resulting in the expression of cleaved-PARP. Thus, cleaved-PARP is considered an important marker of apoptosis [38–40]. The expression of pro-apoptotic protein Bax was increased in the shikonin-treated cells, whereas the expression of the anti-apoptotic protein Bcl-2 was reduced in control cells. Moreover, the expression of cleaved-PARP increased in a shikonin concentration-dependent manner (Figure 3). Therefore, shikonin seemed to induce apoptosis by increasing the expression of Bax and cleaved-PARP, and inhibiting the expression of Bcl-2 protein.

MAPKs are serine/threonine kinases that are responsive to extracellular stimuli. Proteins involved in the MAPK pathway can be largely divided into ERK1/2, JNK, and p38 kinases, and regulate a number of physiological functions [24]. ERK1/2 is primarily involved in cell survival and proliferation; it inhibits apoptosis in response to stress and is induced by stimuli such as tumor necrosis factor (TNF) [41] and radiation [42]. However, overexpression of ERK1/2 by DNA-damage stimuli, such as UV rays, induces apoptosis [43]. Although apoptosis induction through ERK activation has not been clearly elucidated, up-regulation of p53 by activated ERK during apoptosis has been demonstrated to promote apoptosis, depending on the cell line and stimuli [44]. In view of this evidence, Western blotting was performed to determine whether apoptosis induced by shikonin is mediated by the MAPK pathway. A significant increase was observed in the expression of p-ERK1/2, p-JNK, and p-p38 in the shikonin-treated cells compared with that in the control cells (Figure 4). Therefore, apoptosis induction by shikonin in A375SM melanoma cells appeared to be mediated by the expression of ERK and JNK proteins in the MAPK pathway, specifically by the expression of p38.

To determine whether the *in vitro* anticancer effect of shikonin, is extended to *in vivo* conditions, xenograft tumors were grown in mice. Although a significant reduction in tumor tissue volume was observed over the shikonin administration period (Figure 5A), no such reduction in tumor weight was observed after the completion of administration (Figure 5B). TUNEL assay was performed to determine whether the volume reduction could be associated with apoptosis, and the number of TUNEL-positive cells was found to be significantly increased in the shikonin-treated mice compared with that in the control mice (Figure 6A). A previous study investigating the anticancer effect of shikonin treatment on cancer cells reported that apoptosis was induced via the p-p38 pathway [45]. In the present study, examination of changes in p-p38 expression in tumor cells by immunohistochemical staining showed an increased number of p-p38-positive cells in the shikonin-treated mice compared with that in the control mice (Figure 6B). These results indicated that apoptosis was induced due to increased expression of p-p38 in tumor cells, and increased shikonin dosage or longer administration period seemed to eventually result in reduction in tumor volume and weight.

In conclusion, the results of the present study demonstrated that shikonin inhibited the proliferation of A375SM melanoma cells by inducing early and late apoptosis. Apoptosis was induced by increased expression of Bax and cleaved-PARP and decreased expression of Bcl-2 proteins. Apoptosis induction by shikonin was associated with MAPK pathway proteins, ERK, JNK, and p38, *in vitro* and *in vivo*. Therefore, our findings indicate that shikonin, a natural flavonoid, has potential as an anticancer agent against A375SM melanoma.

## Data Availability

All data included in the present study are available upon request by contacting the corresponding author.

## Abbreviations

Bax: Bcl2-associated X protein
Bid: BH3-interacting domain death agonist
Bim: BCL-2 interacting mediator of cell death
DAB: 3,3′-diaminobenzidine
DAPI: 4′,6-diamidino-2-phenylindole
DMEM: Dulbecco’s modified Eagle’s medium
DMSO: dimethylsulfoxide
ERK: extracellular signal-regulated kinase
FBS: fetal bovine serum
H&E: Hematoxylin and Eosin
JNK: c-Jun N-terminal kinase
MAPK: mitogen-activated protein kinase
MTT: 3-(4,5-dimethylthiazol-2-yl)-2,5-diphenyltetrazolium bromide
PARP: poly (ADP-ribose) polymerase
PI: propidium iodide
PS: phosphatidylserine
p-p38: phosphorylated p38
rTdT: Terminal Deoxynucleotidyl Transferase, Recombinant
TNF: tumor necrosis factor
TUNEL: terminal deoxynucleotidyl transferase dUTP nick-end labeling
UV: ultraviolet
